# Index markers of chronic fatigue syndrome with dysfunction of TCA and urea cycles

**DOI:** 10.1038/srep34990

**Published:** 2016-10-11

**Authors:** Emi Yamano, Masahiro Sugimoto, Akiyoshi Hirayama, Satoshi Kume, Masanori Yamato, Guanghua Jin, Seiki Tajima, Nobuhito Goda, Kazuhiro Iwai, Sanae Fukuda, Kouzi Yamaguti, Hirohiko Kuratsune, Tomoyoshi Soga, Yasuyoshi Watanabe, Yosky Kataoka

**Affiliations:** 1 Department of Physiology, Osaka City University Graduate School of Medicine, 1-4-3 Asahimachi, Abeno-ku, Osaka 545-8585, Japan; 2 Institute for Advanced Biosciences, Keio University, 246-2 Mizukami Kakuganji, Tsuruoka Yamagata 997-0052, Japan; 3 Cellular Function Imaging Team, Division of Bio-function Dynamics Imaging, RIKEN Center for Life Science Technologies, 6-7-3 Minatojima-minamimachi, Chuo-ku, Kobe, Hyogo 650-0047, Japan; 4 Hyogo Children’s Sleep and Development Medical Research Center, Hyogo Rehabilitation Centre Central Hospital, 1070 Akebono-cho, Nishi-ku, Kobe, Hyogo 651-2181, Japan; 5 Department of Life Science and Medical Bio-Science, School of Advanced Science and Engineering, Waseda University, 2-2 Wakamatsu-cho, Shinjuku-ku, Tokyo 162-8480, Japan; 6 Department of Molecular and Cellular Physiology, Graduate School of Medicine, Kyoto University, Yoshida-Konoe-cho, Sakyo-ku, Kyoto 606-8501, Japan; 7 Department of Health Science, Kansai University of Welfare Sciences, 3-11-1 Asahigaoka, Kashihara, Osaka 582-0026, Japan; 8 Department of Endocrinology, Metabolism and Molecular Medicine, Osaka City University Graduate School of Medicine, 1-4-3 Asahimachi, Abeno-ku, Osaka 545-8585, Japan; 9 Pathophysiological and Health Science Team, RIKEN Center for Life Science Technologies, 6-7-3 Minatojima-minamimachi, Chuo-ku, Kobe, Hyogo 650-0047, Japan

## Abstract

Chronic fatigue syndrome (CFS) is a persistent and unexplained pathological state characterized by exertional and severely debilitating fatigue, with/without infectious or neuropsychiatric symptoms, lasting at least 6 consecutive months. Its pathogenesis remains incompletely understood. Here, we performed comprehensive metabolomic analyses of 133 plasma samples obtained from CFS patients and healthy controls to establish an objective diagnosis of CFS. CFS patients exhibited significant differences in intermediate metabolite concentrations in the tricarboxylic acid (TCA) and urea cycles. The combination of ornithine/citrulline and pyruvate/isocitrate ratios discriminated CFS patients from healthy controls, yielding area under the receiver operating characteristic curve values of 0.801 (95% confidential interval [CI]: 0.711–0.890, *P* < 0.0001) and 0.750 (95% CI: 0.584–0.916, *P* = 0.0069) for training (n = 93) and validation (n = 40) datasets, respectively. These findings provide compelling evidence that a clinical diagnostic tool could be developed for CFS based on the ratios of metabolites in plasma.

Chronic fatigue syndrome (CFS) is a persistent and unexplained pathological state characterized by exertional and severely debilitating fatigue, with/without muscular, infectious or neuropsychiatric symptoms, lasting at least 6 consecutive months^1^,^2^. CFS patients experience neuropsychological symptoms, including cognitive impairment, chronic widespread pain and depressive symptoms^2^. The pathogenesis of CFS remains incompletely understood and is thought to be multifactorial, including abnormalities of the central nervous system^3^,^4^,^5^, immune system^6^ and the hypothalamo–pituitary–adrenal axis^7^.

To date, the only established criteria for CFS are those from the Centers for Disease Control and Prevention (CDC), which rely on clinical parameters, including patient symptoms and physical examination results^1^. In addition, fatigue severity was evaluated using a performance status scale developed for patients with CFS, the descriptive scale for which ranges from 0 (best performance status) to 9 (worst performance status)^8^,^9^. The inability to objectively and reliably diagnose CFS results in incorrect or delayed diagnosis, which imposes a considerable burden on the patients’ psychological and physical health, as well as economic wellbeing. Because of incomplete understanding of aetiology and diagnostic uncertainty of CFS population, there are no firmly established treatment recommendations for CFS^2^. In practice, therapy, whether pharmacological or nonpharmacological, has been generally directed toward relieving symptoms and improving function^2^. Therefore, the development of objective diagnostic criteria for CFS at an early stage is critical for the development of an effective treatment corresponding to the pathophysiology of the disease.

Previous studies attempted to identify biomarkers^10^,^11^,^12^ or activated viruses including enteroviruses, Epstein–Barr virus and human herpes virus^13^ that could be used for the objective diagnosis of CFS; however, to date, no reliable and economical diagnostics have been established.

Several studies have focused on the metabolic alterations that occur when physical or mental fatigue is induced in healthy subjects^14^,^15^,^16^. When physical fatigue was induced by exhaustive or sustained exercise, the levels of branched-chain amino acids (BCAAs) in the blood were decreased after exercise relative to those before exercise^14^,^15^. In addition, the levels of BCAAs and tyrosine, cysteine, methionine, lysine and arginine were decreased in cases with mental fatigue^16^. However, these findings showed the metabolic alterations induced by a type of acute fatigue in experimental settings in healthy subjects, so they do not reflect the pathophysiology of CFS, which involves exertional and severely debilitating fatigue lasting for at least 6 consecutive months^1^,^2^. Therefore, it is necessary to clarify the specific metabolite profile in CFS to develop an evidence-based strategy for the objective diagnosis and treatment of this disease.

Metabolite profiling provides direct functional information on metabolic phenotypes and indirect functional information on a range of phenotypes that are determined by small molecules, such as disease manifestations 17. Therefore, the comprehensive analysis of metabolites has been used to characterize the pathophysiology of various disease states and thus to assist in drug discovery, disease diagnostics and treatments^17^,^18^. In addition, clarification of the metabolic profile could contribute to the prevention of chronic fatigue caused by metabolic dysfunction via the supply of nutrition in a diet tailored to the individual. We previously validated the utility of plasma metabolomic analysis in a rat model of relatively long-lasting fatigue using capillary electrophoresis mass spectrometry (CE-MS), and found a decrease in energy metabolism with a change in urea cycle metabolism, as well as changes in the levels of amino acids including BCAAs^19^. Other previous studies have reported the amino acid disturbances in CFS using nuclear magnetic resonance (NMR) spectroscopy, which is useful for simplified screening with a limited number of detected metabolites^20^,^21^. These studies had a number of limitations including the small number of subjects involved and lack of validation using multiple cohort datasets.

In the present study, we performed a metabolomic analysis with plasma obtained from CFS patients and healthy controls using CE-MS, which is a highly sensitive method for the detection of metabolites. This approach enabled comprehensive analysis of the metabolic profile in various pathways with high reproducibility. The objectives of our study were to investigate the metabolomic profile of CFS comprehensively, accompanied by a validation process, and to characterize its pathophysiology, with the goal of establishing an objective index for discriminating patients with CFS from healthy controls.

## Results

We analysed the metabolomic profiles of two cohort datasets, namely, training (*n* = 93) and validation data (*n* = 40). Table 1 summarizes the demographic and clinical characteristics of the CFS patients and healthy controls used in this study. Capillary electrophoresis time-of-flight mass spectrometry (CE-TOFMS) successfully identified and quantified 144 metabolites. To identify metabolites that could discriminate CFS patients from healthy controls, we selected metabolites showing large signal/noise ratio (≥10) and few missing values, which resulted in 53 and 33 metabolites in the training and validation data, respectively (Supplementary Fig. S1). Of these, 31 metabolites were consistently observed and used for subsequent analyses.

The statistical significance of differences between CFS patients and healthy controls was assessed. We firstly selected metabolites which showed large margin between CFS and healthy controls, e.g. high discrimination ability using support vector machine–feature selection (SVM-FS), and secondly made indexes using the ratios of two metabolites to discriminate the two groups. Then, a multiple logistic regression (MLR) model using these indexes was developed using training data and validated using the independently collected cohort datasets.

**Figure 1:**
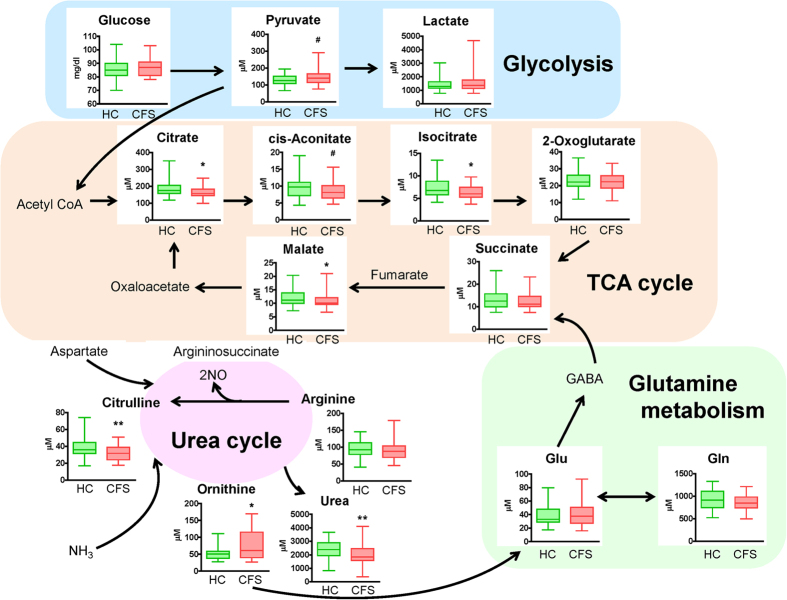
Metabolic pathway map of quantified metabolite concentrations, including for glycolysis, the tricarboxylic acid cycle, the urea cycle and glutamine metabolism, in chronic fatigue syndrome (CFS) patients and healthy controls (HCs). Box-and-whisker plots of the concentrations of metabolites involved in energy metabolism in the plasma of HCs and CFS patients. The coloured plots denote HCs (green) and CFS patients (red). The horizontal lines indicate the minimum, maximum, median, and first and third quartile. ^#^*P* < 0.10; *P < 0.05; **P < 0.01 (Mann–Whitney *U*-test).

**Figure 2:**
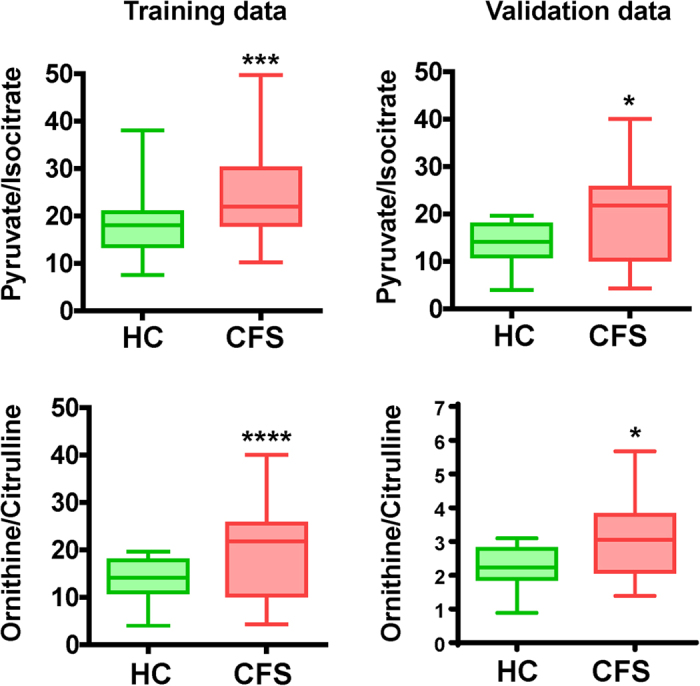
Box-and-whisker plots of ratios of pyruvate/isocitrate and ornithine/citrulline in training and validation datasets. The horizontal lines indicate the minimum, maximum, median, and first and third quartile. *P < 0.05; ***P < 0.001; ****P < 0.0001 (Mann–Whitney *U*-test).

Representative metabolites from the training data were visualized in pathway forms (Fig. 1). No significant difference was observed in glucose concentration when comparing the two groups. In glycolysis, the median concentration of pyruvate of CFS patients tended to increase (*P* < 0.10) relative to that of healthy controls. In the first steps of the tricarboxylic acid (TCA) cycle, the concentrations of organic acids in CFS patients showed significant decreases relative to those in healthy controls, such as citrate (*P* < 0.05) and isocitrate (*P* < 0.05). *cis*-Aconitate in CFS patients tended to decrease (*P* < 0.10) relative to that in healthy controls. The following metabolites in the TCA cycle, malate significantly decreased (*P* < 0.05) in CFS patients. Consequently, the ratio of pyruvate/isocitrate was significantly higher in CFS patients than in healthy controls (*P* < 0.01), reflecting a disturbance in the link between glycolysis and the TCA cycle. In the urea cycle, compared with those in healthy controls, there were significant decreases in the concentrations of urea (*P* < 0.01) and citrulline (*P* < 0.01) and a significant increase in the concentration of ornithine (*P* < 0.05) in CFS patients. Consequently, the ratio of ornithine/citrulline, reflecting metabolic activity in the urea cycle, was significantly higher in CFS patients than in healthy controls (*P* < 0.001). The differences in these ratios between CFS patients and healthy controls were consistent between the training and validation datasets (Fig. 2).

In glutamine metabolism, glutamate and glutamine did not show a significant difference between the two groups (Fig. 1 and Supplementary Table S1). The BCAAs including valine, leucine and isoleucine in CFS patients did not show any significant difference relative to those in healthy controls.

**Figure 3:**
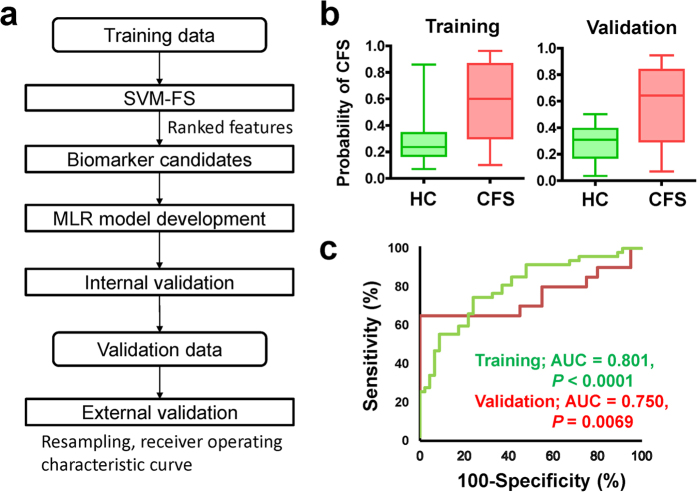
Overall flow of the development and validation of the multiple logistic regression model and results predicted by this model. (**a**) The training data were used for development and internal validation of the model, starting from support vector machine-feature selection, feature selection based on metabolic pathways, model development and internal validation. The model predicted independent validation datasets that were not used for model training. (**b**) Box-and-whisker plots of the probability of chronic fatigue syndrome yielded by the developed model. Horizontal lines indicate the minimum, maximum, median and first and third quartile. (**c**) Receiver operating characteristic curves for training and validation datasets.

SVM-FS ranked ornithine, citrulline, lactate, isocitrate and pyruvate as the top five metabolites for discriminating CFS patients from healthy controls (Supplementary Table S2). Of these, ornithine and citrulline are the substrate and product of ornithine carbamoyltransferase [EC: 2.1.3.3] in the urea cycle, respectively, so we used the ornithine/citrulline ratio as an index to assess the activity of this pathway. In a similar manner, the pyruvate/isocitrate ratio was also used as another index to assess the activity of glycolysis and the TCA cycle. An MLR model combining these indexes showed area under receiver operating characteristic (ROC) curve (AUC) values of 0.801 [95% confidence interval (CI): 0.711–0.890, *P* < 0.0001] and 0.750 (95% CI: 0.584–0.916, *P* = 0.0069) for training and validation datasets, respectively. A flowchart on the development of the MLR model and the predicted results are shown in Table 2 and Fig. 3. As a rigorous validation, 200 trials of 10-fold cross validation (CV) yielded a high median value of AUC values: 0.784 (95% CI: 0.783–0.785), and 200 resampling trials also yielded high AUC values: 0.790 (95% CI: 0.783–0.796) and 0.758 (95% CI: 0.741–0.768) for the training and validation datasets, respectively. These results indicated that the combination of ornithine/citrulline and pyruvate/isocitrate ratios clearly discriminated CFS patients from healthy controls.

## Discussion

In this study, metabolomic analyses of CFS patients and healthy controls revealed that the concentrations of organic acids related to the TCA cycle and energy metabolism, such as citrate, isocitrate and malate, were significantly lower in CFS patients than in healthy controls, although the concentration of glucose did not differ significantly between the two groups. As for the urea cycle, the concentration of ornithine in CFS patients was significantly higher than in healthy controls, while that of citrulline was significantly lower in CFS patients than in healthy controls. The metabolites in glutamine metabolism and the BCAAs did not show significant differences between the CFS patients and the healthy controls. The decreased concentrations of organic acids related to the TCA cycle and energy metabolism in the CFS patients suggested that they have deficiencies in adenosine triphosphate (ATP) production secondary to dysregulation of the flow from pyruvate to citrate via acetyl CoA and abnormalities in the conversion of citrate to isocitrate by aconitase. This profile was thought to reflect the pathophysiology of energy metabolism in CFS. Since more than 90% of cellular energy is produced from the electron transport chain, which is a process of ATP generation using O_2_ and electron donors (such as NADH_2_^+^) in mitochondria^22^, the decreases in the concentrations of organic acids involved in the TCA cycle suggest a deficiency of ATP production in CFS patients^23^,^24^,^25^. Aconitase activity is closely associated with ATP production; the inhibition of aconitase reduces the cellular energy supply^26^,^27^ and induces cell death^28^. Aconitase includes a 4Fe–4S cluster and is vulnerable to oxidative stress^29^,^30^,^31^,^32^. In addition to the previous study^33^, the findings that CFS patients who were recruited from the same hospital as the present study showed higher oxidative stress relative to healthy controls^34^ supports the hypothesis that the predominant reduction of isocitrate may result from the inactivation of aconitase via chronic oxidative stress. Indeed, muscle pain, which is one of the major symptoms of CFS, was also reported to be induced by oxidative stress^29^. The decreased concentrations of metabolites in the first steps of the TCA cycle in CFS patients likely reflect the pathophysiology of fatigue, which shows the functional dysregulation of flow from pyruvate to isocitrate. Thus, the plasma pyruvate to isocitrate ratio might be an appropriate marker of this type of dysregulation.

As for the BCAAs, they did not show any significant difference in CFS relative to healthy controls. We previously reported that a fatigued animal model showed increases in BCAAs including valine, leucine and isoleucine, which could be induced by the proteolysis of skeletal muscle upon deprivation of physical rest^32^. Considering this, CFS might not involve sustained muscle stress.

A previous study using NMR metabolomics for CFS reported an increase of blood glucose and lactate as well as a decrease in urine pyruvate and alanine in CFS patients, suggesting a possible inhibition of glycolysis by the reduced provision of adequate amounts of acetyl-CoA required for the citric acid cycle^21^. There were some differences between these studies and ours, probably due to the different analytical methods used, i.e. NMR and CE-MS detect different types of metabolites based on their chemical properties^35^. The severity of disease in subjects with CFS is another factor that might cause different alterations of metabolites. Despite these discrepancies, the previous study indicated that dysfunctional energy metabolism through the citric acid cycle is a fatigue phenotype^21^. This hypothesis is in accord with our findings showing the functional dysregulation of flow in the TCA cycle might reflect the pathophysiology of fatigue.

Our findings can be attributed to a distinctive metabolic pathway in the fatigued condition: the metabolic flow from ornithine and glutamine to succinate of the TCA cycle via gamma-aminobutyric acid (GABA)^36^,^37^. Increased ornithine in the urea cycle in the fatigued condition may cause upregulation of the flow of glutamate into the TCA cycle via GABA and the succinate pathway. In the present study, the concentrations of citrate and isocitrate in the first steps of the TCA cycle related to the energy metabolism in CFS patients were reduced relative to those in healthy controls. Meanwhile, the concentration of succinate, which follows these metabolites in the TCA cycle, did not differ significantly between CFS patients and healthy controls. This suggested that activation of the fatigue metabolic pathway contributes to the supply of energy that has been reduced under the fatigued condition.

A previous study reported that the ornithine/citrulline ratio is a marker that can be used to evaluate the activity of the urea cycle^38^. An increase in the ornithine/citrulline ratio indicates a relative slowdown at the entry point of the urea cycle where ornithine is combined with carbamyl phosphate by ornithine transcarbamylase to make citrulline, which is the first intermediate in this cycle after the rate-limiting enzyme carbamyl phosphate synthetase I^39^. Our results also showed that the ornithine/citrulline ratio was significantly higher in CFS patients than in healthy controls. This might reflect the enzymatic dysfunction of either carbamyl phosphate synthetase I, which makes carbamyl phosphate from ammonia and HCO_3_, or ornithine transcarbamylase^38^ in CFS. Ornithine and citrulline, which is produced from ornithine and carbamyl phosphate by ornithine transcarbamylase, are involved in the major function of detoxification in the liver^40^. A previous study reported that citrulline is also produced from arginine by the NO production pathway and is catalysed by nitric oxide synthase, which is upregulated by AMP-activated protein kinase in response to ADP/ATP imbalance^41^. The metabolic balance of citrulline and arginine is reported to influence intracellular and extracellular lipid peroxidation levels^42^,^43^. In fatigued animals, a previous study showed that plasma NO_X_ levels were increased, and plasma oxidative levels were also elevated^19^. Another study reported that thiobarbituric acid-reactive lipoperoxide was increased in the liver tissue of fatigued animals^44^. Taking these findings together, the higher oxidative stress levels in the plasma and serum in CFS^33^,^34^ may cause metabolic dysregulation, such as the inactivation of aconitase, which likely results in the reduction of isocitrate in the TCA cycle and the inhibition of metabolic flow from ornithine to citrulline in the urea cycle.

With AUC values of 0.801 (95% CI: 0.711–0.890, *P* < 0.0001) and 0.750 (95% CI: 0.584–0.916, *P* = 0.0069) for the training and validation datasets, respectively, the two ratios of pyruvate/isocitrate and ornithine/citrulline levels in plasma could be used to distinguish between CFS patients and healthy controls. Both CV and resampling validations showed narrow 95% CIs, indicating the low dependence of the predictive performance on the given data and confirming the model’s versatility. AUC values of a single index (i.e. pyruvate/isocitrate or ornithine/citrulline) were 0.709 (95% CI: 0.601–0.816, *P* = 0.00053) and 0.758 (95% CI: 0.660–0.856, *P* < 0.0001) for training data, respectively, and 0.705 (95% CI: 0.526–0.884, *P* = 0.027) and 0.695 (95% CI: 0.521–0.870, *P* = 0.035) for validation data, respectively, which were less than the AUC values using the two indexes combined. Therefore, we suggest that these two ratios represent objective index markers that can facilitate rapid screening for CFS.

In the present study, metabolomic analysis revealed decreased activity in the TCA cycle and the urea cycle in CFS patients. Application of the two ratios, pyruvate/isocitrate and ornithine/citrulline, which might reflect such dysregulation mainly in these two cycles in CFS patients, specifically discriminated between CFS patients and healthy controls. Because our findings may reflect the pathophysiological state of CFS, they might contribute not only to the objective diagnosis but also to the treatment of CFS patients by indicating appropriate nutrients to be ingested in food or supplements.

There were several limitations in this study. Additional studies with a larger patient population should be performed to confirm the metabolic dysregulation in the TCA cycle and the urea cycle, which are thought to reflect the pathophysiological state in CFS. The developed discrimination model should be further validated using a larger population. In addition, longitudinal measurements with detailed clinical investigations are needed to prove its generalizability. To discriminate CFS patients from healthy controls, it is possible that other combinations of quantified metabolites can also be used. However, the use of the ratio of two metabolites as an index, rather than an individual metabolite concentration, would eliminate the inconsistency in overall concentration derived from diurnal variation. In this study, we only utilized the data identified by our standard library whereas TOF-MS provides non-targeted data. The peaks without assignment, but which showed potential discriminating ability, should also be analysed in future studies to identify other markers and provide a more accurate discrimination. The number of peaks showing S/N≥10 was different between the training and validation data (Supplementary Fig. S1). Although the metabolites were quantified by eliminating the bias of MS sensitivity, other factors, such as sprayer setting and splay condition, might also affect the sensitivity of the observed data. More rigorous quality control should be implemented in future studies.

Time-course analyses of metabolomic profile in individual patients for short and long periods might help to realize the diagnosis with higher accuracy.

In summary, our study has demonstrated a profile of abnormal energy metabolism resulting from deficiencies in aconitase activity in the TCA cycle and dysregulation in the urea cycle in CFS patients. Two ratios, pyruvate/isocitrate and ornithine/citrulline, the changes of which may reflect inactivity of the two above-mentioned cycles in CFS, could be useful index markers to discriminate CFS patients from healthy controls. Although a further large-scale investigation is needed, the metabolite index markers identified in this study provide compelling evidence that a clinical diagnostic tool could be developed for CFS based on the ratios of small molecules in plasma.

## Methods

### Subjects

The subjects in this study were 133 Japanese adults, namely, 67 CFS patients and 66 healthy controls. A total of 47 CFS patients and 46 healthy controls were assigned to the training set, while 20 CFS patients and 20 healthy controls were included in the validation set. Patients ranging in age from 20 to 60 years old were recruited at the Fatigue Clinical Centre of Osaka City University Hospital (Osaka, Japan). Healthy controls ranging in age from 20 to 60 years old were recruited via online advertisements and shift workers were excluded. A diagnosis of CFS was made for individual patients by the staff at the Fatigue Clinical Centre, in accordance with the criteria proposed by the CDC^1^. Subjects with psychiatric disorders or with chronic diseases that are sometimes accompanied by fatigue (e.g., cancer, diabetes) and those taking medications known to affect autonomic nerve function or the central nervous system were excluded. All subjects with neuropsychiatric disorders were diagnosed by doctors of general medicine, neurology and psychiatry at Osaka City University Hospital. The protocol was approved by the Institutional Review Board and was conducted in accordance with the Declaration of Helsinki. All subjects provided written informed consent for participation in this study.

### Biological sample processing of human materials

Plasma samples were collected from subjects in the training set between 9:00 a.m. and 12:00 p.m. All samples were collected from April 2012 to December 2012. For those in the validation set, samples were collected from fasting subjects (>2 h without any food or nutritious drink) from September 2006 to December 2009. To extract metabolites, 40 μl of plasma was placed in 400 μl of methanol containing 20 μM each of methionine sulfone, 2-(N-morpholino)ethanesulfonic acid and D-camphor-10-sulfonic acid, and the preparation was mixed well. Then, 120 μl of deionized water and 400 μl of chloroform were added, and the solution was centrifuged at 10,000 × *g* for 3 min at 4 °C. The upper aqueous layer was filtered via centrifugation through a Millipore 5-kDa cut-off filter at 9,100 × *g* for 120 min at 4 °C to remove large molecules. Then, the solution was concentrated by centrifugation for 3.5 h at 45 °C, and samples were lyophilized until needed for CE-TOFMS analyses^45^,^46^. For metabolite analysis, samples were dissolved in 50 μl of Milli-Q water containing 200 μM each of 3-aminopyrrolidine and trimesic acid for CE-TOFMS.

### Metabolomics

All CE-TOFMS experiments were performed using an Agilent CE capillary electrophoresis system (Agilent Technologies, Waldbronn, Germany), Agilent 1669A Accurate-Mass TOF LC/MS system (Agilent Technologies, Palo Alto, CA, USA), Agilent 1100 and 1200 series isocratic high-performance LC pump, G1603A Agilent CE-MS adapter and G1600AX Agilent CE electrospray ionization (ESI)-MS sprayer kit. For anion analysis, an Agilent G7100-60041 platinum ESI needle was used. Agilent ChemStation software (ver. A.10.02, B.02.01.SR1, and B.03.02) for CE and Agilent MassHunter software (ver. B.02.00 and B.02.02) were used for system control and data acquisition.

### CE-TOFMS conditions for cationic metabolite analysis

Separations were carried out in a fused silica capillary (50 mm i.d. × 100 cm total length) filled with 1 M formic acid as the electrolyte. Approximately 3 nl of sample solution was injected at 50 mbar for 5 s, and voltage of 30 kV was applied. The capillary temperature was maintained at 20 °C and the sample tray was cooled below 5 °C. Methanol-water (50% v/v) containing 0.1 μM hexakis(2,2-difluoroethoxy)phosphazene was delivered as the sheath liquid at 10 μl/min. ESI-TOFMS was performed in the positive ion mode and the capillary voltage was set at 4,000 V. The flow rate of heated dry nitrogen gas (heater temperature, 300 °C) was maintained at 10 psig. In TOFMS, the fragmentor, skimmer and octapole radio frequency voltages (Oct RFV) were set at 75, 50 and 125 V, respectively. Automatic recalibration of each acquired spectrum was performed using the masses of reference standards ([^13^C isotopic ion of a protonated methanol dimer (2MeOH+H)]^+^, *m*/*z* 66.063061) and {[hexakis(2,2-difluoroethoxy)phosphazene +H]^+^, *m*/*z* 622.028963}. Exact mass data were acquired at a rate of 1.5 spectra/s over the *m*/*z* range of 50–1,000^45^,^46^.

### CE-TOFMS conditions for anionic metabolite analysis

A commercially available COSMO (+) capillary (50 mm i.d. × 102 cm; Nacalai Tesque, Kyoto, Japan), chemically coated with cationic polymer, was used as the separation capillary. A 50 mM ammonium acetate solution (pH 8.5) was used as the electrolyte solution for CE separation. Sample solution (30 nl) was injected at 50 mbar for 30 s, and a voltage of 30 kV was applied. Ammonium acetate (5 mM) in 50% methanol–water (v/v) containing 0.1 μM hexakis was delivered as the sheath liquid at 10 μl/min. ESI-TOFMS was conducted in the negative ion mode; the capillary voltage was set at 3,500 V. For TOFMS, the fragmentor, skimmer and Oct RFV voltages were set at 100, 50 and 200 V, respectively. Automatic recalibration of each acquired spectrum was performed using the masses of reference standards ([^13^C isotopic ion of deprotonated acetic acid dimer (2CH3COOH-H)]^−^, *m*/*z* 120.038339), and ([hexakis + deprotonated acetic acid (CH3COOH-H)]^−^, *m*/*z* 680.035541). Exact mass data were acquired at a rate of 1.5 spectra/s over the *m*/*z* range of 50–1,000. Other conditions were identical to those used in the cationic metabolite analyses^45^,^46^.

### Data processing and statistical analysis

Raw CE-TOFMS data were analysed using our proprietary software, MasterHands^47^, which follows typical data processing flows, including the detection of all possible peaks, elimination of noise and redundant features, and generation of an aligned data matrix^48^. The detailed algorithms and parameters, such as (1) peak piking and integrating peak area, (2) correcting migration times of individual peaks using a dynamic programming approach, and (3) matching peaks among multiple datasets to generate a data matrix, were described previously^49^. The metabolite names of our standard compound library were assigned for each peak by matching corrected migration times and *m*/*z* values. To calculate the metabolite concentration, peak areas of detected peaks were divided by a peak area of each internal standard (methionine sulfone and D-camphor-10-sulfonic acid for cations and anions, respectively) to eliminate fluctuation of the sensitivity of mass spectrometry. Standard mixtures including these internal standards were also measured and metabolite concentrations of each sample were calculated based on the ratio of these relative peak areas.

The Mann–Whitney *U-*test was used to assess the statistical significance of differences between CFS patients and healthy controls. Demographic data were compared between the groups using Student’s *t*-test and categorical data were compared using Fisher’s exact test. A value of *P* < 0.05 was considered statistically significant. Statistical analyses were performed using the R statistics package (R Foundation for Statistical Computing, Vienna, Austria; www.r-project.org) and IBM SPSS for Windows ver. 22.0 (IBM, Armonk, NY).

To select metabolites with a strong potential for discriminating CFS patients from healthy controls, metabolites showing a high signal/noise ratio (≥10) in both training and validation datasets and with few missing values were selected. Among these metabolites, those consistently observed in both training and validation data were used as biomarker candidates. SVM-FS was used to rank the discrimination ability of these biomarker candidates; among the top metabolites, two (a substrate and a product) were selected based on their metabolic pathway and used for indexes to assess the activity within the part of the pathway between the two metabolites. We applied this approach utilizing the ratios of two metabolites to eliminate diurnal variation in the background concentration of metabolites.

An MLR model was developed using these indexes. This was conducted using training validation datasets and the developed model predicted independent validation datasets (e.g., different cohort data). The discrimination ability of the model was assessed by ROC analysis and its AUC value. The versatility of the model was validated using 200 trials of 10-fold CV as follows. (1) The datasets were randomly split into groups comprising 90% and 10% of the individuals. (2) The former and the latter were used for model development and validation, respectively. (3) These procedures were repeated up to 10 times, and the accuracy of the model was evaluated using the predictions of validation data. To eliminate optimistic bias in the prediction, we also conducted 200 trials of resampling and 10-fold CV as follows. (1) Virtual datasets were yielded by selecting individuals, allowing redundant selection (resampling). (2) A prediction model was developed for each dataset. Overall analytical flow is shown in Fig. 3a. Missing values were replaced by half of the minimal value of each metabolite for each set of data (as described elsewhere^50^ with slight modification). These analyses were conducted using JMP (ver. 11.2.0; SAS, Cary, NC), WEKA (ver. 3.6.0; University of Waikato, Hamilton, NZ; www.cs.waikato.ac.nz/ml/weka) and GraphPad Prism (ver. 5.04; GraphPad Software Inc., San Diego, CA).

## Additional Information

**How to cite this article**: Yamano, E. *et al.* Index markers of chronic fatigue syndrome with dysfunction of TCA and urea cycles. *Sci. Rep.* **6**, 34990; doi: 10.1038/srep34990 (2016).

## Supplementary Material

Supplementary Information

## Figures and Tables

**Table 1:** Demographic and clinical characteristics of the study subjects.

	Training dataset			Validation dataset		
	Healthy controls (n = 46)	CFS patients (n = 47)	*P* value	Healthy controls (n = 20)	CFS patients (n = 20)	*P* value
Age (years)	38.78 ± 9.71	38.08 ± 6.57	0.35	36.10 ± 8.35	36.15 ± 8.14	0.99
Sex (F/M)	41/5	41/6	0.78	10/10	10/10	1.0
Performance status	—	5.57 ± 1.64	—	—	5.75 ± 1.86	—
BMI	19.93 ± 4.90	20.98 ± 3.62	0.96	21.96 ± 2.82	21.03 ± 2.60	0.29
Glucose (mg/dl)	85.56 ± 7.65	87.00 ± 5.93	0.52	89.71 ± 10.84	90.85 ± 10.31	0.73

Values are expressed as mean ± SD or number/number. *P* values were obtained by Student’s *t*-test or Fisher’s exact test.

**Table 2:** MLR model.

Metabolite	Parameter	95% CI		Odds ratio	95% CI		*P* value
Pyruvate/Isocitrate	−0.128	−0.200	−0.067	0.880	0.819	0.935	0.000
Ornithine/Citrulline	−0.705	−1.16	−0.314	0.494	0.312	0.730	0.001
(Intercept)	4.41	2.82	6.28	—	—	—	<0.0001
