# Supplementary material for: Index markers of chronic fatigue syndrome with dysfunction of TCA and urea cycles
Source: Sci Rep. 2016 Oct 11;6:34990. doi: 10.1038/srep34990 (PMC5057083; doi:10.1038/srep34990)
Supplement: Supplementary Information [file srep34990-s1.pdf]

Supplementary Information for

Index markers of chronic fatigue syndrome with dysfunction of TCA and urea cycles

Emi Yamano, Masahiro Sugimoto, Akiyoshi Hirayama, Satoshi Kume, Masanori Yamato, Guanghua Jin, Seiki Tajima, Nobuhito Goda, Kazuhiro Iwai, Sanae Fukuda, Koji Yamaguti, Hirohiko Kuratsune, Tomoyoshi Soga, Yasuyoshi Watanabe, Yosky Kataoka*

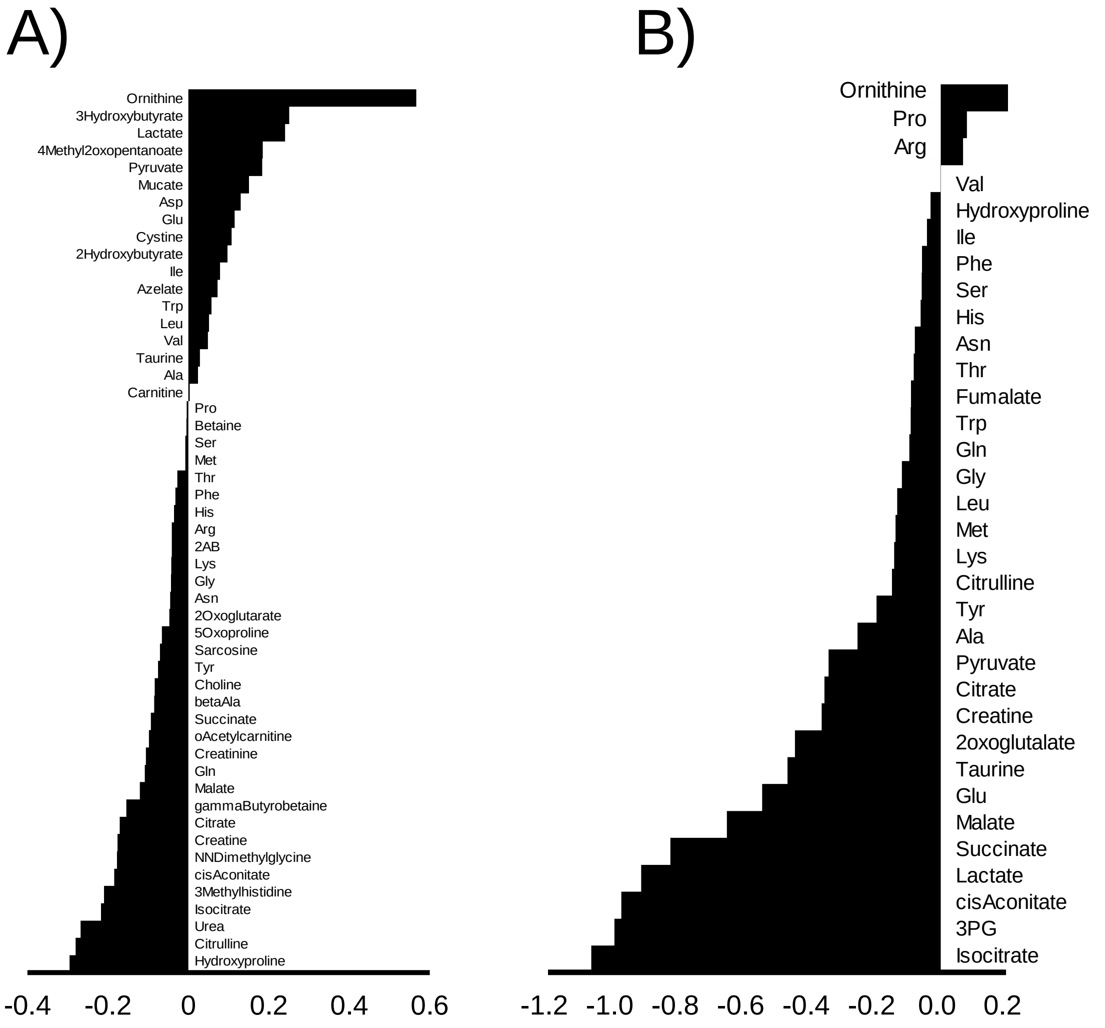

Figure S1. Log$_2$ fold changes of the mean concentration between CFS patients and healthy controls for training data **A)** and validation data **B)**.

Table S1 Comparisons of glucose and representative metabolite concentrations between CFS patients and healthy controls in the training cohort.

Name	Healthy controls		CFS patients		*P* value	Corrected *P* value
	Mean	SD	Mean	SD		
Glucose	85.57	7.65	87.00	5.93	0.52	0.59
Pyruvate	130.43	30.52	148.16	47.27	0.087	0.16
Lactate	1375.06	394.35	1624.85	839.54	0.49	0.59
Citrate	183.63	40.98	163.10	36.03	0.010	**0.049**
cis-Aconitate	9.74	3.15	8.57	2.64	0.080	0.16
2-Oxoglutarate	23.13	5.38	22.39	5.17	0.63	0.63
Succinate	13.24	4.05	12.41	3.59	0.30	0.46
Malate	12.00	3.01	11.04	3.16	0.047	0.12
Arg	94.31	23.98	91.64	30.66	0.39	0.54
Urea	2426.33	622.12	2015.10	686.34	0.0010	**0.012**
Ornithine	52.15	16.36	77.25	42.24	0.023	0.083
Citrulline	38.50	11.91	31.70	8.31	0.0030	**0.026**
Glu	37.59	13.71	40.71	17.38	0.55	0.59
Gln	917.43	218.58	851.15	178.37	0.16	0.27

The unit of glucose is mg/dl; the others are µM.

Mann–Whitney *U*-test was performed.

Significant corrected *P* values with a false discovery rate ($P < 0.05$) are in bold type.

SD, standard deviation.

Table S2. Ranking using SVM-FS

Rank	Metabolite
1	Ornithine
2	Citrulline
3	Lactate
4	Isocitrate
5	Pyruvate
6	Succinate
7	Hydroxyproline
8	Taurine
9	Tyr
10	Val
11	Gln
12	Citrate
13	Trp
14	Ala
15	Glu
16	Leu
17	Phe
18	Creatine
19	Ile
20	Arg
21	Thr
22	*cis*-Aconitate
23	Pro
24	2-Oxoglutarate
25	Gly
26	Malate
27	His
28	Lys
29	Ser
30	Asn
31	Met

SVM-FS; Support vector machine–feature selection.
